# Ilomastat, a synthetic inhibitor of MMPs, prevents lung injury induced by γ-ray irradiation in mice

**DOI:** 10.18632/oncotarget.18487

**Published:** 2017-06-15

**Authors:** Xiaoman Li, Dehui Ma, Xiaodan Zha, Dongqin Quan, Dong Pan, Manji Sun, Burong Hu, Baoquan Zhao

**Affiliations:** ^1^ State Key Laboratory of Toxicology and Medical Countermeasures, Institute of Pharmacology and Toxicology, Academy of Military Medical Sciences, Beijing, China; ^2^ CAS Key Laboratory of Heavy Ion Radiation Biology and Medicine, Institute of Modern Physics, Chinese Academy of Sciences, Lanzhou, China; ^3^ Key Laboratory of Space Radiobiology of Gansu Province, Lanzhou, China; ^4^ College of Animal Science and Technology, Inner Mongolia University for Nationalities, Tong Liao, China; ^5^ University of Chinese Academy of Sciences, Beijing, China

**Keywords:** ilomastat, radiation-induced lung injury, pneumonitis, lung fibrosis, MMPs, Pathology Section

## Abstract

Lung injury is one of the pathological features in human or animal after radiation and the main side effect for patient after lung cancer radiotherapy. The efficient protective strategy still needs to exploit and the underlying mechanisms remain to be investigated. We found that the expression and activity of matrix metalloproteinases (MMPs) significantly increased at the early stage of radiation-induced lung injury (RILI). Pretreatment with Ilomastat, a synthetic inhibitor of MMPs, decreased the expression and activity of MMPs and significantly alleviated the lung inflammation and fibrosis in the irradiated mice, as well as enhanced the survival of irradiated mice. In addition, the levels of TGF-β, IL-6, TNF-α and IL-1β in the tissues dramatically reduced in the irradiated mice pretreated with Ilomastat. Furthermore, our experiments *in vitro* also showed that radiation significantly increased the MMPs activity, and Ilomastat pretreatment inhibited the activity of MMPs activated by irradiation and increased the cell survival. It is the first report, to our knowledge, to demonstrate that Ilomastat is a potential effective reliever for RILI and MMPs may play important roles in the process of RILI.

## INTRODUCTION

In the recent decades, nuclear technology application such as nuclear medicine and nuclear power station has a robust development over time. Correspondingly, the possibility of exposure to radiation for nuclear workers and patients greatly rise. In addition, with the rapid development of manned space technology, the health and safety of the astronauts are being paid more and more attention because of the impact of space radiation environment. Lung is prone-sensitive to ionizing radiation (IR) and one of the most common radiation toxicities is radiation induced pneumonitis and fibrosis, namely radiation-induced lung injury (RILI). Radiotherapy is a main treatment for pulmonary tumors [1], which confines the dose escalation for increasing the effectiveness of radiotherapy. The percentage of radiation induced pneumonitis after thoracic irradiation was reported to be at a matter of 15%, and parts of these patients died from radiation pneumonitis [1]. The method to reduce the risk of this severe radiation pneumonitis is to control the dose of radiation or the volume of lungs that are exposed to the radiation fields. Although there has several medical therapies to slow the progression of pulmonary fibrosis, the side effects are inevitable and these therapies do not effectively reduce the mortality [2, 3]. Herein, it is urgent to further explore a safe and effective biological radioprotector.

MMPs are a family of enzymes that degrade components of the extracellular matrix. In normal physiology condition, MMPs lowly express in the majority of normal tissues, except for those circumstances of matrix remodeling and wound healing. Among these MMPs, MMP2 and MMP9 play important roles in extracellular basement membrane turnover by preferentially degrading type IV collagen, a main component of the basement membrane, therefore, affecting the structural integrity of the lung tissue [4, 5]. MMPs is capable of interacting with a variety of substrates, particularly during an inflammatory response. More importantly, MMPs can facilitate the inflammation cytokines activation, for example, TGF-β, IL-1β and TNF-α. Those cytokines reversely activate the preproenzyme, which forms a positive loop to aggravate lung damage [6-8]. Additionally, MMPs also have been implicated in the development of fibrosis, but their precise role has not been determined [9-14]. Several studies suggest that inhibition or deletion of MMPs could alleviate the development of fibrosis [5, 10-12]. Evidence also showed that recombinant tissue inhibitor of metalloproteinases 2 (rTIMP-2) suppressed immune complex induced high permeability pulmonary edema in rats and that a synthetic MMPs inhibitor protected rat lungs from oxidant-induced injury [15]. Overexpression of MMPs is not only associated with the invasion and angiogenesis of multiple types of tumors, but also involved in many inflammatory disease including chronic obstructive pulmonary disease (COPD) and multiple sclerosis (MS) that are believed to be impeded by inhibiting this gelatinase [7].

Ilomastat (HONHCOCH2CH (i-Bu) CO-L-Trp-NHMe; GM6001, Galardin) is a broad spectrum inhibitor of MMPs. It binds to the critical active-site zinc atom of this class of proteinases, which has broad antitumor and anti-angiogenic activity [16]. Ilomastat is also been used in trauma healing, and cornea repair [17-19]. Human clinical trials for ophthalmic applications of Ilomastat have been conducted without reported toxicities [16]. However, study on its protective effect on RILI is not available yet. In this study, we sought to explore if this lung injury could be prevented by Ilomastat, and whether and which MMPs play an important role in the development of RILI in mouse. A series of experiments carried out by us suggest that radiation induced MMPs overexpression aggravates RILI, and this lung injury could be prevented by Ilomastat.

## RESULTS

### Irradiation induces the upregulation of MMPs’ activity and Ilomastat pretreatment decreases it

**Figure 1:**
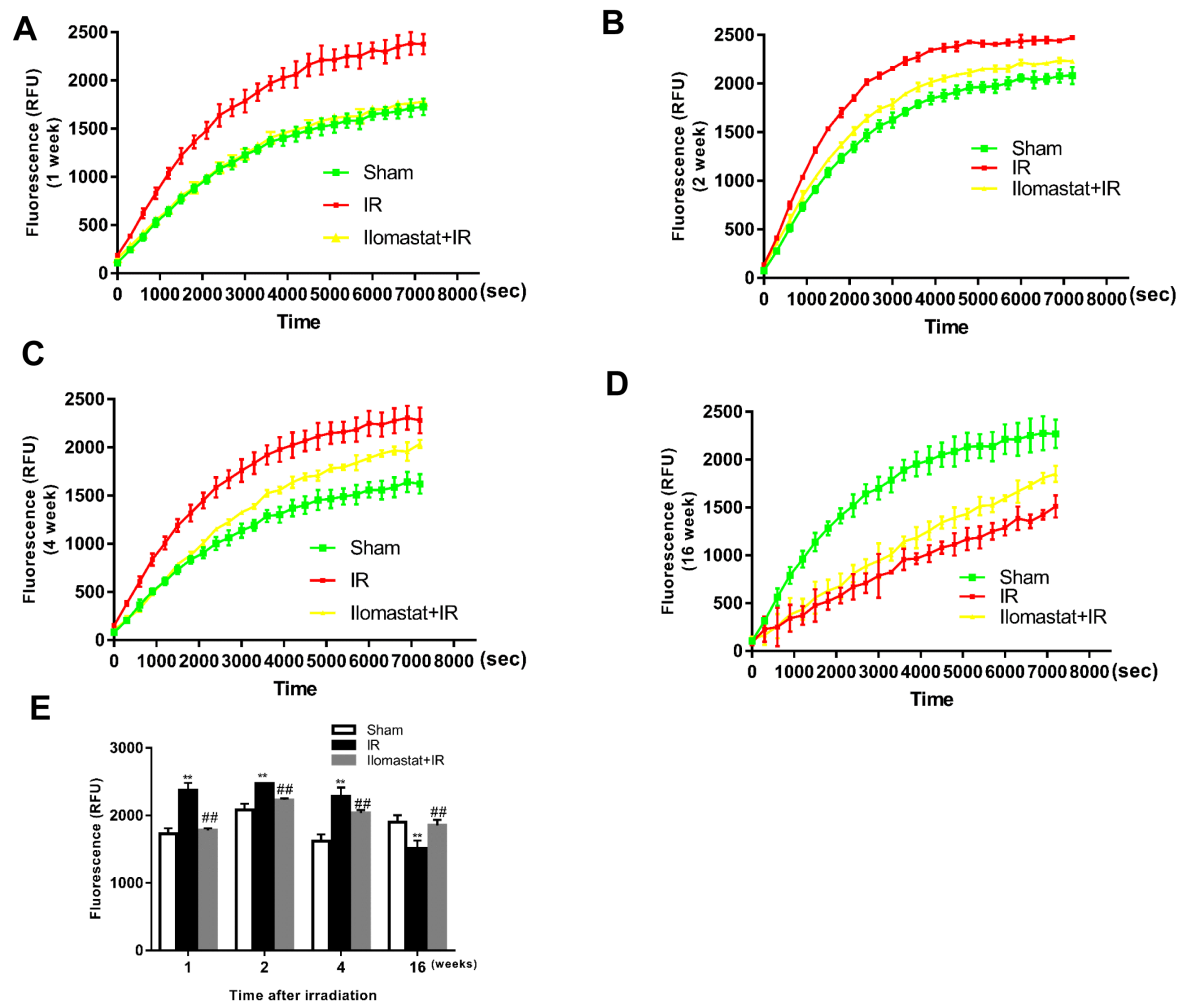
Total activity of MMP proteases in lung tissues of mice after irradiation A fluorogenic substrate, Mca-KPLGL-Dap (Dnp)-AR-NH_2_, was added to the lung tissue homogenate at a final concentration of 5 mM in assay buffer to detected the MMPs activity in lung tissues of mice at 1 w **A.**, 2 w **B.**, 4 w **C.** and 16 w **D.** after irradiation. The value of fluorescence (RFU) at 120 min of each treatment group at 1, 2, 4 and 16 w are made histogram to compare the change of total activity of MMPs after irradiation **E.**. Bars represent mean ± SD from three individuals. **P < 0.01 *vs.* the sham control group; *##P* < 0.05 *vs.* the irradiated group.

Firstly, MMPs protease activity in the lung tissue homogenate of mice after irradiation was confirmed by fluorescence enzymatic activity assay using a fluorescence resonance energy transfer (FRET) peptide, Mca-KPLGL-Dap (Dnp)-AR-NH_2_, as the MMPs substrate. In the intact FRET peptide, the fluorescence is quenched because of one part neighboring the other part of peptide. Upon cleavage into two separate fragments by MMPs, the fluorescence is recovered. We detected the relative fluorescence units (RFU) from 0 to 120 min for the samples at 1, 2, 4 and 16 w after irradiation to determine the MMPs activity (Figure 1A, 1B, 1C & 1D). We found that irradiation significantly increased the hydrolysis activity of MMPs and MMPs inhibitor (Ilomastat) pretreatment decreased the hydrolysis activity during 1 to 4 w (Figure 1E). However, at the end of 16^th^ w, the MMPs activity in irradiated group was lower than the sham group and Ilomastat pretreatment group (*P* < 0.05), which is similar to the report that the overall expression of the MMPs was very lower with the fibrosis progress [4].

### Ilomastat inhibits the expression of MMP2 and MMP9

**Figure 2:**
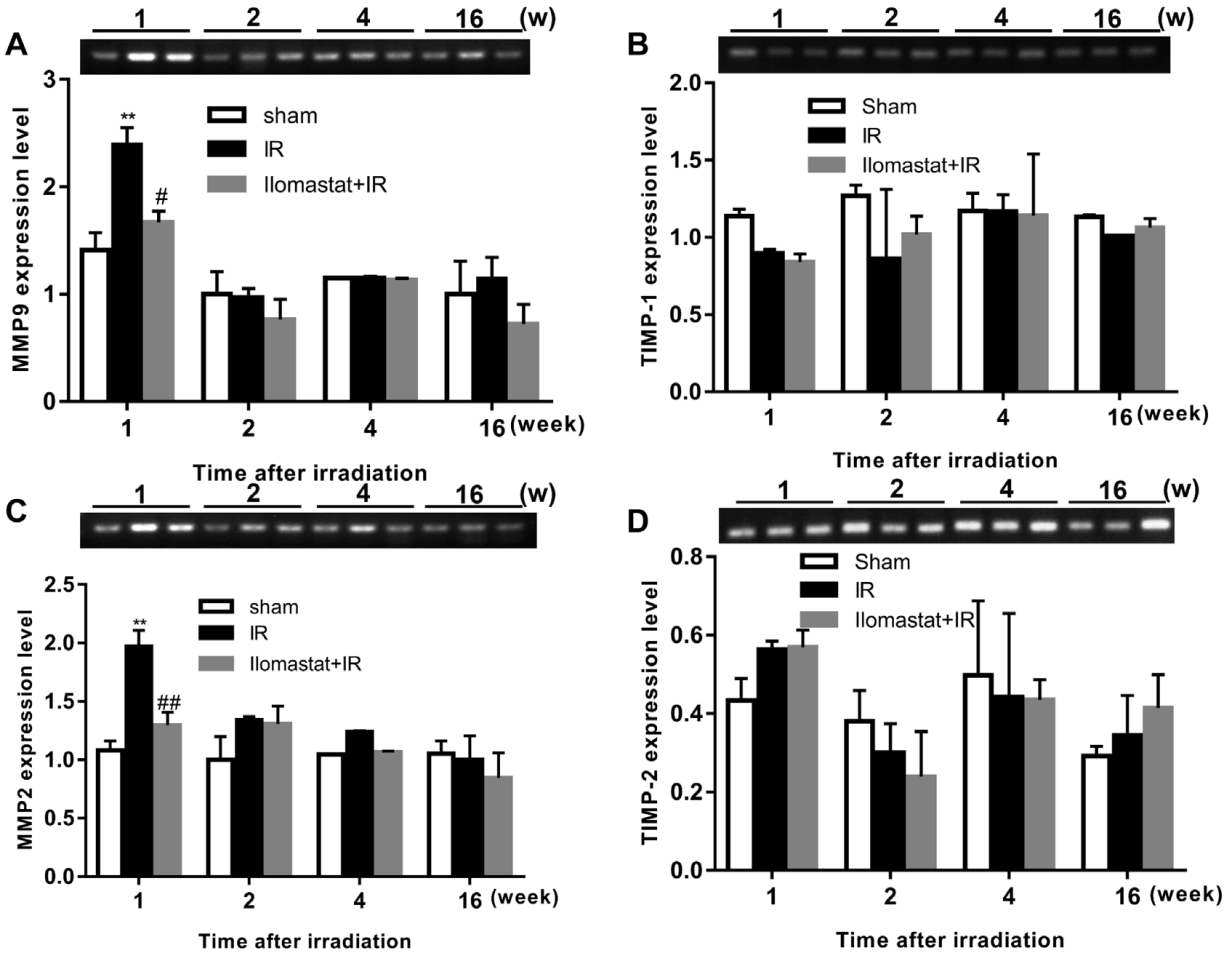

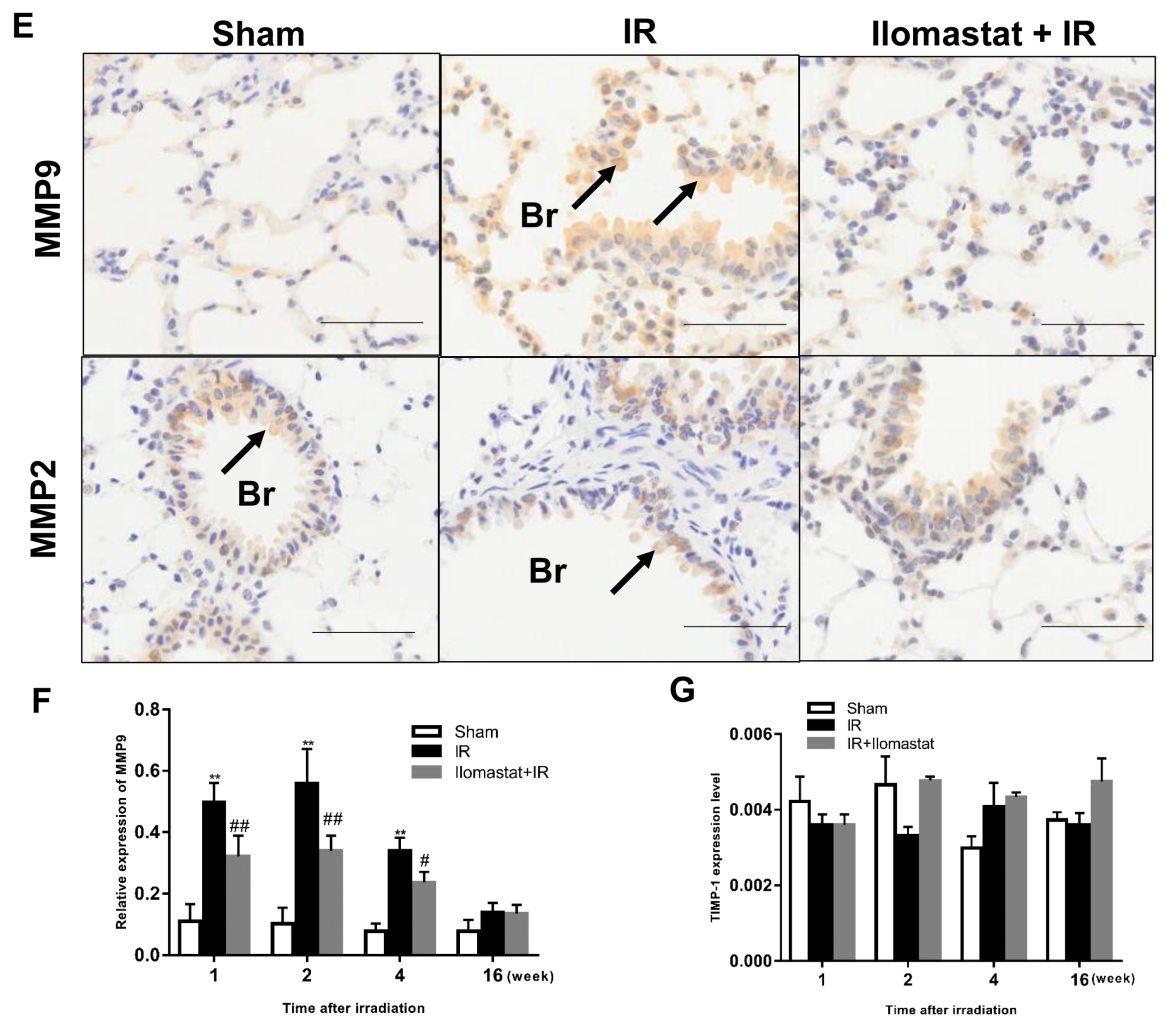
Effect of Ilomastat on the expressions of MMPs and TIMPs in lung tissues The expressions of MMP9 **A.**, MMP2 **B.**, TIMP-1 **C.** and TIMP-2 **D.** mRNA in the lung tissues of mice after sham treatment, 15 Gy thorax irradiation or pretreatment with Ilomastat combined 15 Gy thorax irradiation were detected using semi-quantative RT-PCR. The products of the PCR were electrophoresed on a 1.5 % agarose gel and photographed. The representative images were shown in the upper of each statistics chart. Data are mean ± SD of three different mice. ^*^P < 0.05 *vs*. sham group; ^#^*P* < 0.05 *vs*. irradiation group. **E.** The expressions of MMP9 and MMP2 proteins measured by the immunohistochemical analysis in lung tissue. Representative images of immunohistochemical staining of MMP9 (upper pannel) and MMP2 (lower pannel) in lung tissue obtained at the end of the 2^nd^ w after thoracic irradiation (Black arrows indicate the positive cells stained by MMP9 or MMP2 primary antibody). Br presents the bronchiole. Scale bars, 50 μm. **F.** The relative expression of MMP9 protein in the lung tissue of the mice at different time after irradiation was calculated and demonstrated with histogram. Data are mean ± SD from three mice. ^**^P < 0.01 *vs*. the sham control group; ^##^*P* < 0.05 *vs*. the irradiated group. **G.** Relative expression level of TIMP-1 was measured by qRT-PCR. β-actin was used as internal control.

Since MMP9 and MMP2 are believed to have pivotal roles in RILI [20], we investigated whether Ilomastat can decrease the expression of MMP9, MMP2, and their natural inhibitors TIMP-1 and TIMP-2 in mice. Molecular level analysis of MMP2 and MMP9 mRNA expression using semi-qPCR (in all genes compared with β-actin) showed a significant induction of these two MMPs at the end of the 1^st^ w after 15 Gy irradiation (Figure 2A & 2B) with the exception of TIMP-1 and TIMP-2 expression (Figure 2C & 2D). After treatment with Ilomastat for 2 h before radiation, the expressions of MMP2 and MMP9 presented only a slightly increase compared to the sham control group (Figure 2A & 2B). This indicates that Ilomastat treatment significantly decreased the MMP2 and MMP9 mRNA expression in mice. However, there had no significant difference in the mRNA expression of MMP2 and MMP9 between different groups at the end of the 2^nd^, 4^th^ and 16^th^ w (Figure 2A & 2B). Radiation has no significant influence on the mRNA expression of TIMP-1 and TIMP-2 (Figure 2C & 2D) measued by semi-qPCR. To confirm the TIMP-1 expression, qRT-PCR assay was also used and the similar trend indicated by semi-qPCR was still observed that radiation can not induce the significant increase of TIMP-1 expression (Figure 2G).

In addition, we investigated the expression of MMP9 and MMP2 proteins in tissue. The representative immunohistochemistry images of MMP9 and MMP2 expression in the sham control, Ilomastat pretreatment combined radiation and radiation alone mice are shown in Figure 2E. In the sham control group, MMP2 and MMP9 expressed in moderate degrees in the bronchiolar epithelial cells and weakly expressed in a few alveolar macrophages and type II pneumocytes, as well as in some lymphocytes around the arterioles and bronchioles. In the IR groups, however, intense immunoreactivity was observed throughout the entire course in the bronchiolar epithelial cells (Figure 2F). The inflammatory cells were also strongly positive. Whereas, the expression of MMP2 appeared at a lower level in the experimental period compared to MMP9 (Figure 2E, lower panel). Positive MMP9 and MMP2 expression was identified primarily in the cytoplasm of pneumocyte, especially in type II pneumocyte (Figure 2E). The MMP9 also appeared in the intraalveolar edema fluid which was most prominent at the end of the 4^th^ w. MMP9 started to be stained strongly in pneumocyte from the 1^th^ w, most intensely at the end of the 2^nd^ w (Figure 2F). The integrative expression dramatically increased from the 1^st^ w, reached the peak at the end of the 2^nd^ w. From the 4^th^ w, they slightly diminished, but still remained at significantly higher levels till the end of the 16^th^ w, compared to the sham control group (*P* < 0.05) (Figure 2F). In contrast, Ilomastat decreased the expression of MMP9 in these cells. From the 4^th^ w, there were fewer inflammatory cells, and the intensity of the MMP9 staining weakened.

### Ilomastat effectively attenuates the pneumonitis induced by IR

**Figure 3:**
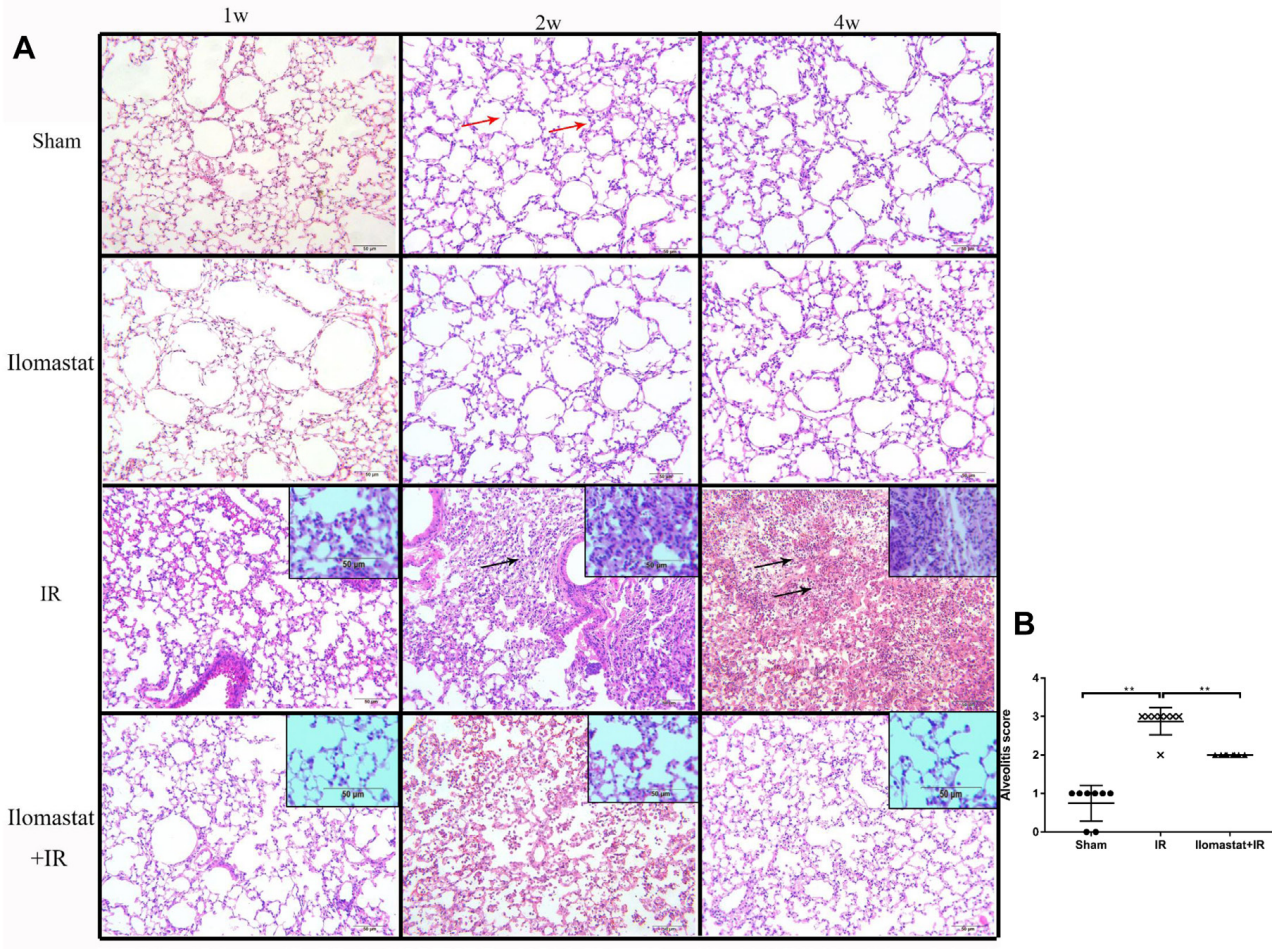
Development of the inflammation in the lung tissues after irradiation or Ilomastat pretreatment followed by irradiation **A.** Histologic changes of the inflammation in the lung tissues were shown by H&E staining (original magnification, 100×; inset magnification, 200×). Red arrows point to the normal alveolar structure and black arrows point to the inflammatory cell infiltration and alveolar structural damages. Nuclei: blue; cytoplasm: pink. Insets are magnifications. **B.** Pulmonitis score in the irradiated group, Ilomastat pretreated plus irradiated group and sham control group. Five random, non-overlapping fields of three mice were used for the scoring. Graphic symbols indicate means ± SD areas in each group. ^**^P < 0.01.

Since MMPs play important roles in the development of acute lung injury and lung inflammation is the early stage of lung injury [4], we therefore investigated the effect of the MMPs inhibitor, Ilomastat, on the radiation-induced pneumonitis. As shown in the middle panel of Figure 3, severe pneumonitis was observed at the end of the 4^th^ w in mice after 15 Gy γ-ray irradiation to thorax. There were inflammatory cell infiltration, inflammatory exudate, and alveolar structural damages in particular alveolitis. The severity of pneumonitis was in a time-dependent manner (Figure 3A, the third panel). The pneumonitis were significantly relieved after pretreatment with Ilomastat followed by irradiation (Figure 3A, the fourth panel), compared to the irradiation alone. Besides, there is no difference of the lung tissue change in the sham group among the observation period (Figure 3A, the second panel). The alveolitis score of irradiation group significantly higher than sham group or Ilomastat pretreatment group (Figure 3B, *P* < 0.01). These results suggest that Ilomastat effectively protects the mouse from radiation-induced pneumonitis.

### Ilomastat attenuates the pulmonary leukocyte influx induced by IR

**Figure 4:**
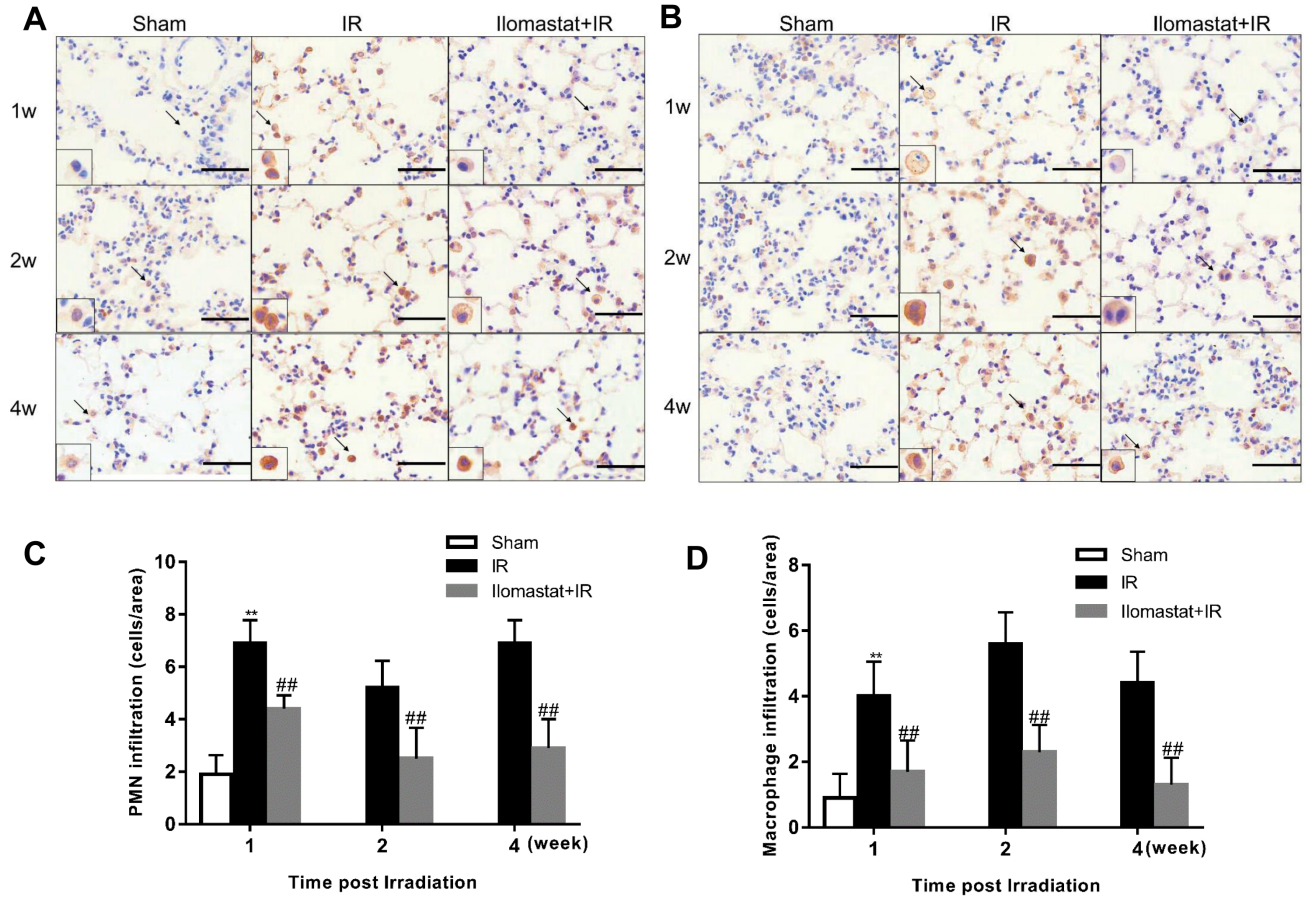
Ilomastat attenuates the pulmonary leukocyte influx induced by irradiation Lung sections of the mice at 1, 2 and 4 w after exposure were immunostained with antibodies of MPO **A.** or F4/80 **B.** to indicate the massive neutrophil and macrophage influx in the lungs of control mice. Arrows indicate polymorphonuclear (PMN) or macrophage in insets. Original magnification, 100×; inset magnification, 200×. Scale bars, 20 μm. Amount of neutrophils **C.** and macrophages **D.** infiltrated in lung sections counted in different areas (*n* = 5) of at least 3 different individuals. Graphic symbols indicate means ± SD areas in each group. ^*^P < 0.05, ^**^P < 0.01 *vs*. sham group; ^#^*P* < 0.05, ^##^*P* < 0.01 *vs*. irradiation group.

As leukocyte infiltration is believed to have an essential role in pneumonitis [21], we studied the role of Ilomastat on the infiltration of leukocytes in the lung after irradiation challenge. We found that both neutrophils (Figure 4A & Figure 4C) and macrophages (Figure 4B & Figure 4D) first appeared in the lung parenchyma as early as 1 w after irradiation challenge and became abundant at later time-points. The leukocyte influx was strongly diminished in Ilomastat pretreated mice (Figure 4C & Figure 4D), which is consistent with the reports that infiltration of these inflammatory cells depends on MMPs and is an essential step towards lethality [22].

### Ilomastat attenuates the pulmonary fibrosis induced by IR

**Figure 5:**
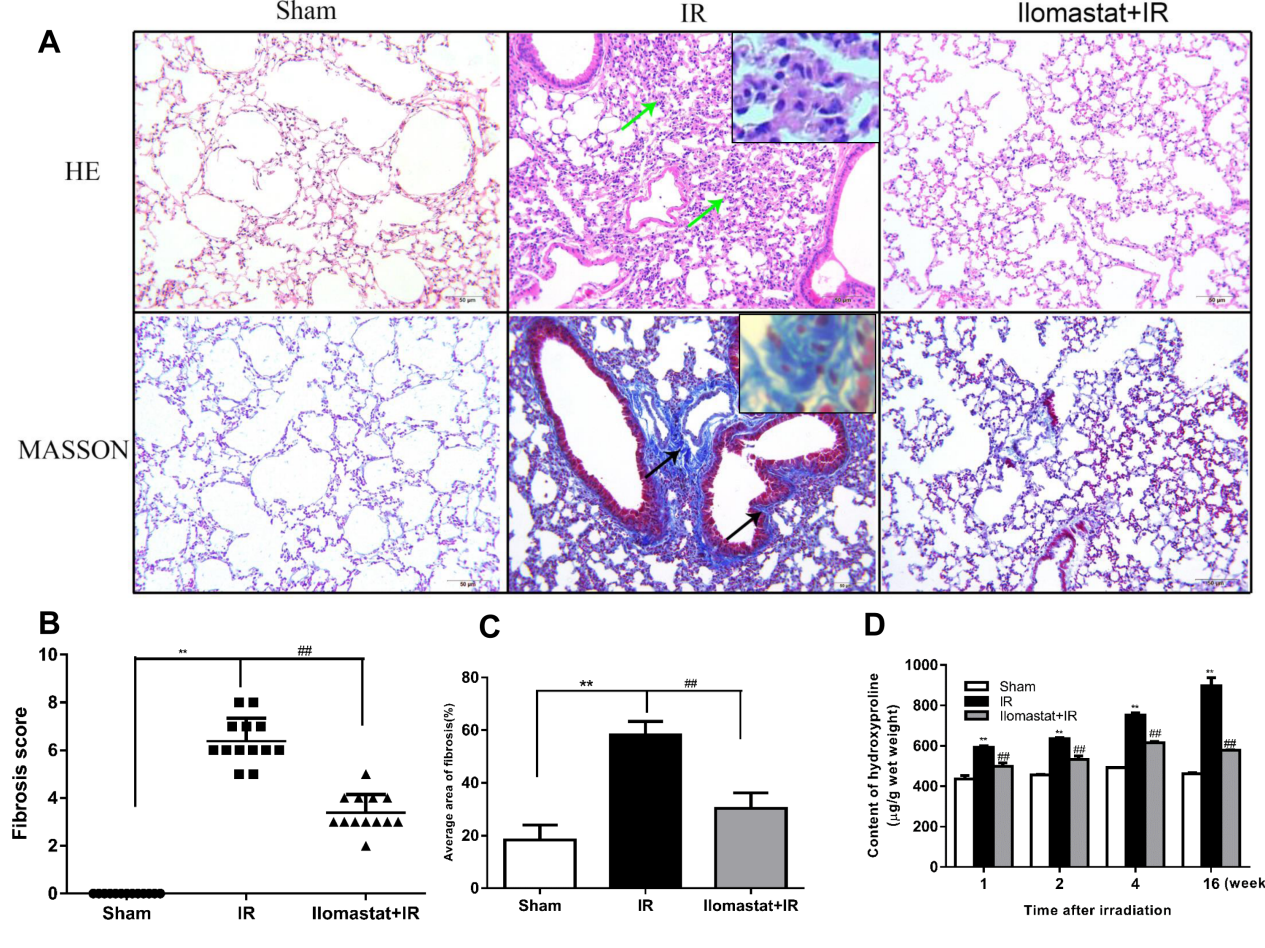
Protective effect of Ilomastat on the pulmonary fibrosis induced by irradiation at the 16th w Histologic changes in the lung tissues were shown by H&E and Masson staining **A.** Green arrows indicate the thickened interalveolar septa and the destruction of alveoli. Black arrows indicate large amounts of collagen depositions (original magnification, 200×). Collagen: blue; nuclei: purple; cytoplasm: pink. Scale bar = 50 µm. Insets are magnifications. Fibrosis scores **B.** and average fibrosis area **C.** of the sham control group, irradiation group and Ilomastat administration group were analysed and Ilomastat administration before radiation significantly decreased the score and average area of lung fibrosis. **D.** Comparison of lung hydroxyproline content among the sham, irradiation and Ilomastat pretreatment groups. Bars indicate the mean ± SD of three to five different mice. ^**^P < 0.01 vs. sham control group. ^##^*P* < 0.01 vs. irradiation group.

The pulmonary fibrosis is the late stage of RILI. Next, we investigated whether inhibition of MMPs can compromise the pulmonary fibrosis or not. The representative histologic changes at the end of the 16^th^ w are shown in Figure 5A. In the lung tissues from the radiation alone group, interalveolar septa became thicken and infiltrated by inflammatory cells, with large amounts of collagen depositions in the interstitium visualized by masson staining (Figure 5A, middle column). For animals pretreated with Ilomastat before radiation, the pathological alteration was relatively mild with a slight thickening of the alveolar septa and the destruction of alveoli appeared minimal (Figure 5A, right column). Whereas, there has no prominent collagen depositions in the sham control group (Figure 5A, left column). The mean Aschcroft fibrosis scores were 3.4 for Ilomastat pretreatment following radiation group and 6.4 for radiation only group, respectively. The score of Ilomastat pretreatment following radiation group was lower than that of the irradiation group (*P* < 0.01, Figure 5B). In addition, the mean areas of fibrosis of each group were 58.2% (IR group), 30.3% (Ilomastat pretreatment following radiation) and 18.3% (sham group), respectively. In the Ilomastat pretreatment following radiation group, there was a greater reduction in the areas of lung fibrosis than in the IR group (*P* < 0.01).

In addition, the concentration of hydroxyproline, an indice of lung fibrosis, in the tissue of mouse after pretreatment with Ilomastat followed by irradiation was also lower than that of the IR group (Figure 5D). At the same time, we found that the concentration of hydroxyproline in tissue increased in a time-dependent manner and reached a peak at end of the 16^th^ w at least in our observation period (Figure 5D). Encouragingly, the concentration of hydroxyproline in tissue kept a lower level in animals pretreated with Ilomastat following radiation. These results suggest that Ilomastat alleviates the lung fibrosis through decreasing collagen deposition.

### Ilomastat decreases the content of cytokines in the lung BALF

**Figure 6:**
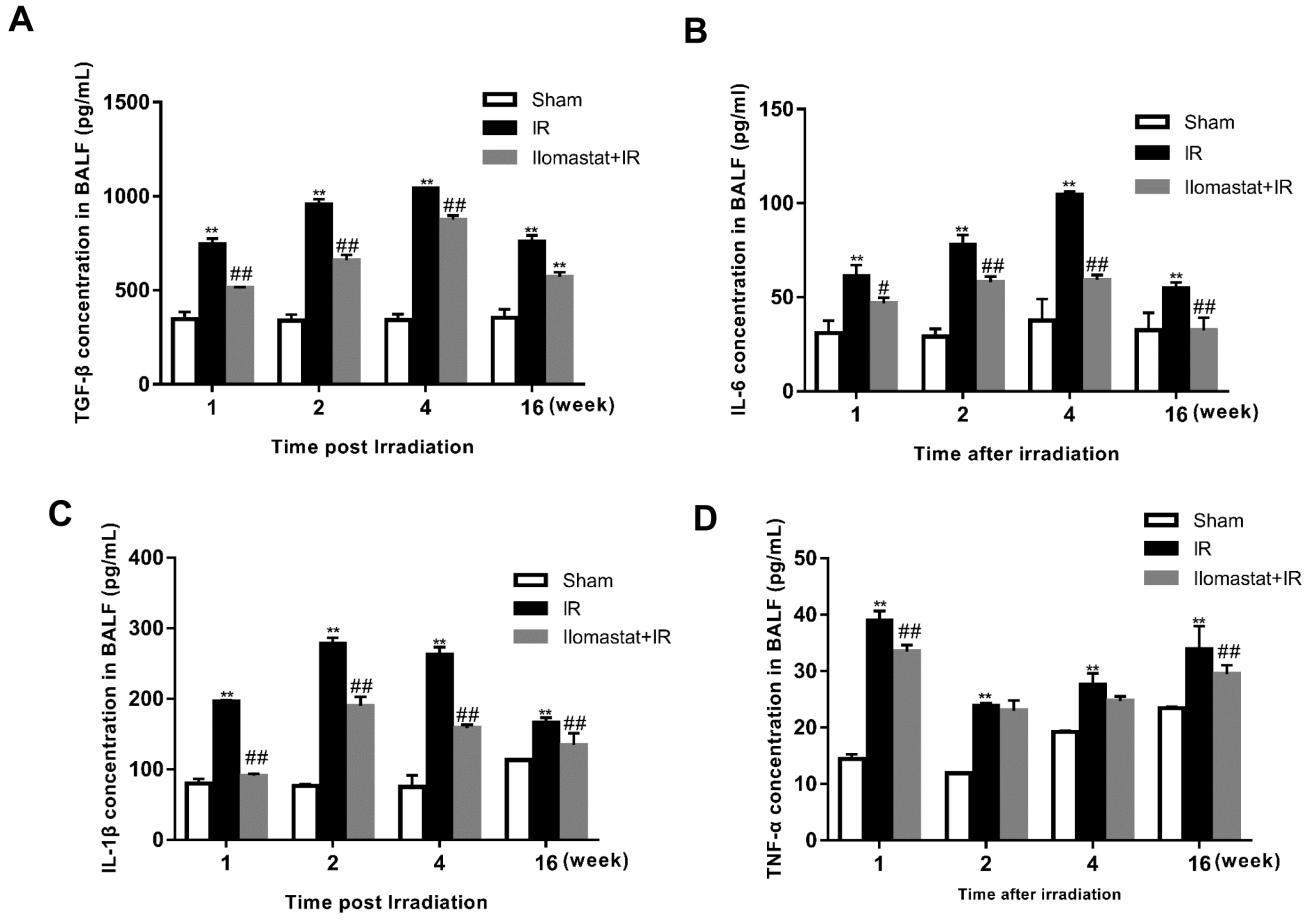
Effect of Ilomastat on the generations of cytokines in the BALF detected by ELISA The lungs removed from mice were homogenized and centrifuged. Supernatants were measured by the ELISA kits of TGF-β **A.**, IL-6 **B.**, IL-1β **C.** and TNF-α **D.** The data were obtained from lungs of four to five mice at per time point and indicate mean values ± SE. ^**^P < 0.01 *vs*. sham group; ^#^*P* < 0.05 and ^##^*P* < 0.01 *vs*. irradiated group.

As previously reported, TGF-β and IL-6 act as the biomarker for RILI [23, 24]. In addition, TNF-α and IL-1β also have important role in RILI. Thus, we investigated the effect of Ilomastat on the generations of TGF-β, IL-6, TNF-α and IL-1β. The concentrations of TGF-β or IL-6 reached the peak at the end of the 4^th^ w post-irradiation and decreased thereafter in radiation group (Figure 6A & 6B). IL-1β reached the peak at the end of the 2^nd^ w post-irradiation (Figure 6C). Whereas, TNF-α reached maximum at the end of 1^st^ w or earlier (Figure 6D). Noting that, TNF-α and TGF-β kept a persistent higher level at the end of 16^th^ (Figure 6A & 6D), which promotes the development of lung fibrosis [25, 26]. After receiving Ilomastat treatment before radiation, the level of TGF-β and IL-6 decreased at the indicated time point after irradiation, which were significantly lower than the corresponding radiation group (*P* < 0.01, Figure 6A & 6B). MMPs may therefore influence the RILI progression through TGF-β, IL-6, TNF-α and IL-1β, and Ilomastat can decrease those cytokine levels to relieve RILI.

### Ilomastat inhibits radiation induced pneumocyte apoptosis

**Figure 7:**
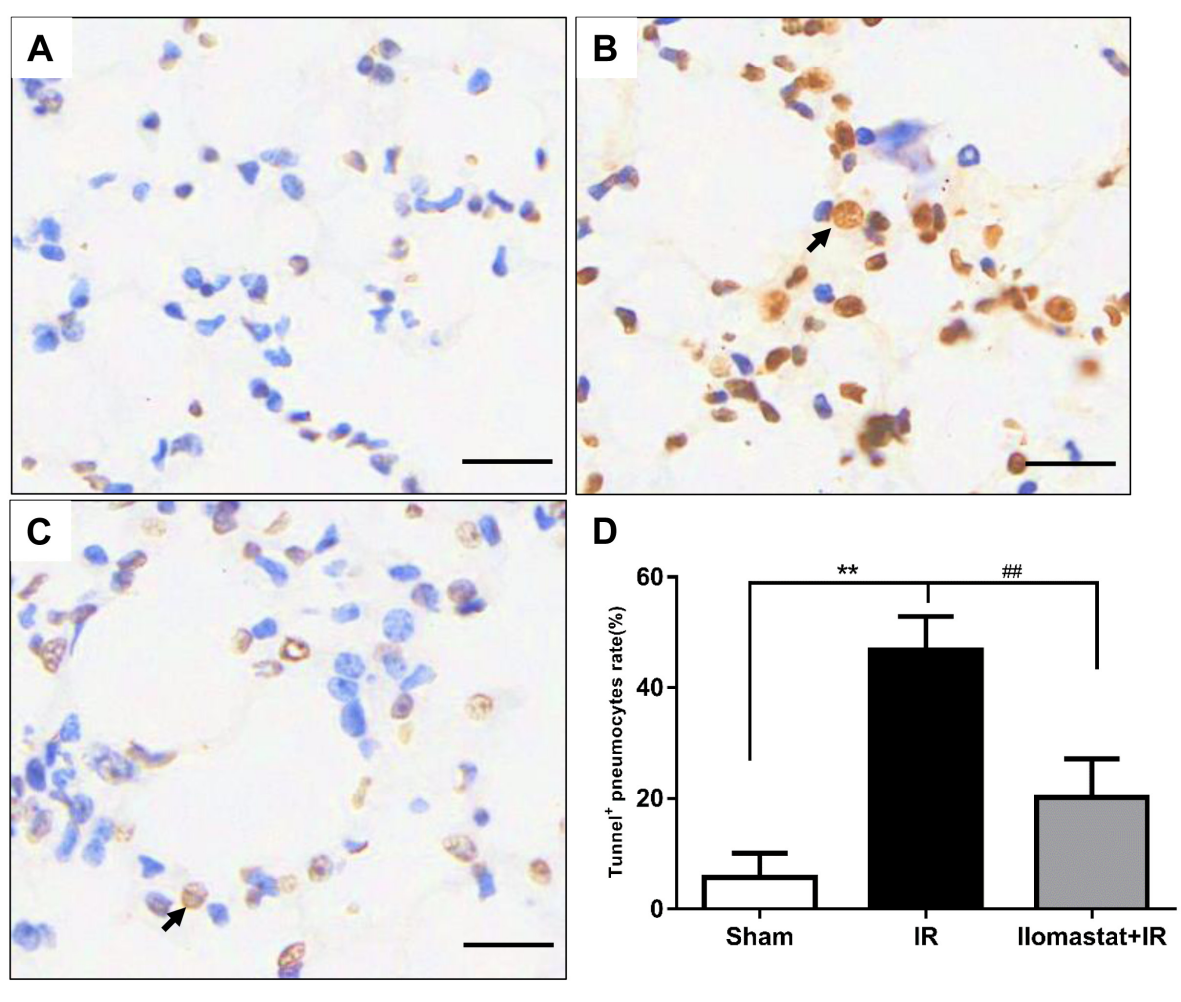
Protective effect of Ilomastat on pneumocyte apoptosis measured by TUNEL Lung tissue sections were prepared 4 w after irradiation or Ilomastat pretreatment followed by irradiation. TUNEL staining were carried out according to the manufacture’s instruction and the representative images were shown (**A.** Sham, **B.** IR, **C.** Ilomastat+IR). The black arrow indicates the TUNEL-positive pneumocyte. Original magnification, 200×, Scale bars, 50 μm. **D.** TUNEL-positive cells rate in the lung tissue sections. Results are expressed as mean ± SD from three to five mice in each group. ^*^P < 0.05 *vs*. sham group; ^#^*P* < 0.05 *vs*. irradiated group.

Inhibition of MMPs gene can alleviate the endothelial cell apoptosis [27]. We therefore investigated the apoptosis in pneumocyte after irradiation and whether Ilomastat can decrease pneumocyte apoptosis through inhibition of MMPs. Terminal deoxyuridine nick-end labeling (TUNEL) staining of the pneumocyte demonstrated that the apoptotic cells dramatically presented compared to the sham or Ilomastat pretreatment following radiation groups (Figure 7B *vs* 7A & 7C). As expected, the apoptosis of pneumocyte significantly decreased at the end of the 4^th^ w for the mice pretreated with Ilomastat following radiation. The fraction of TUNEL-positive cells in radiation group was higher by about two folds than the Ilomastat pretreated mice, and by about five folds than the sham control mice (Figure 7D). These results suggest that Ilomastat also protect lung injury by inhibiting the apoptosis caused by accumulation of MMPs.

### Ilomastat protects the ultrastructures of the lamellar body and mitochondria

**Figure 8:**
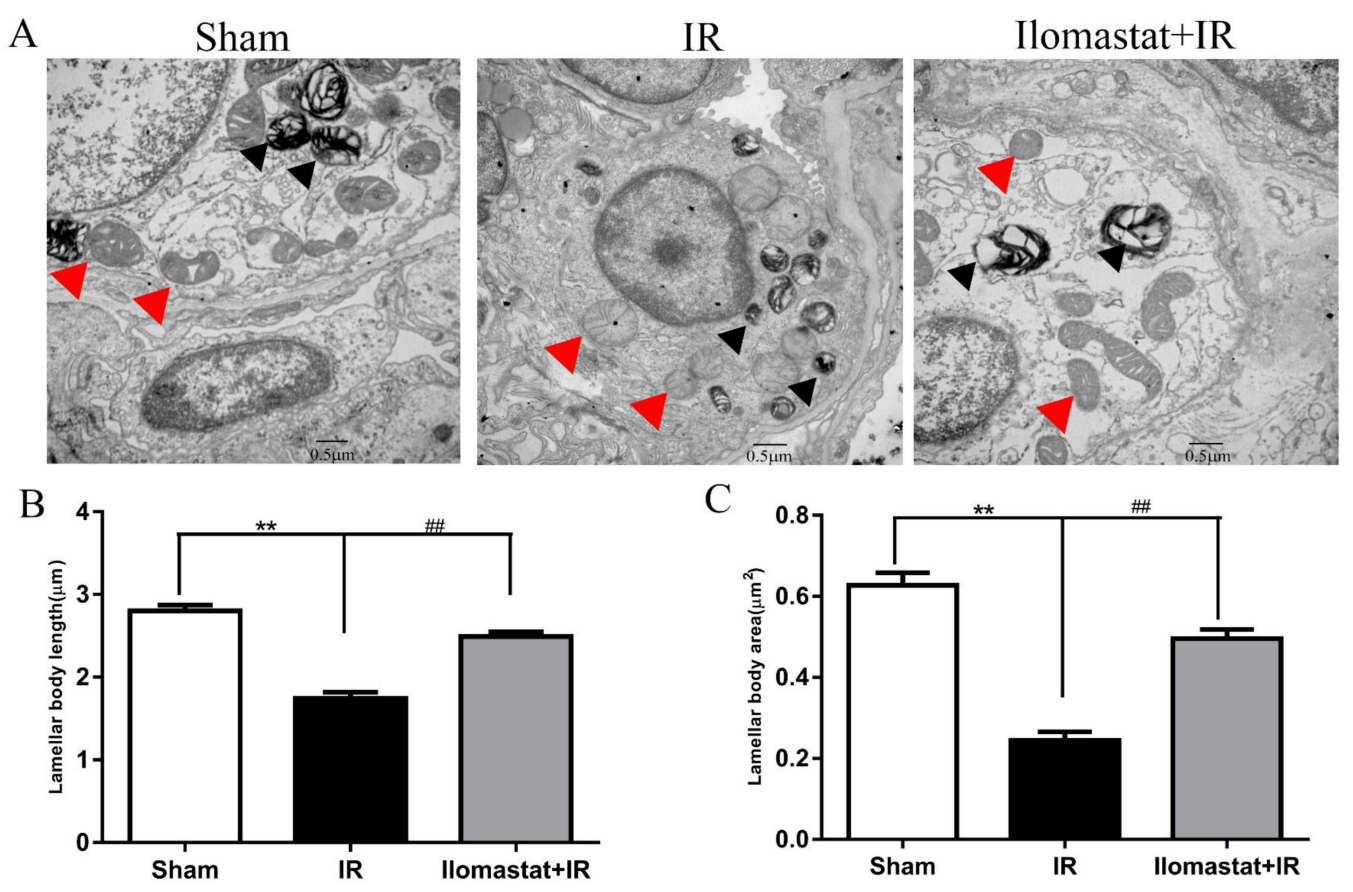
The ultrastructure of the lamellar body and mitochondria of type II pneumocytes in the lung tissue sections Lamellar body and mitochondrial morphology scanned by TEM **A.** At least eight images were taken from each sample at 20,000× magnification and the micrograph is representative of five mice in each group. Lamellar body and mitochondria in the type II pneumocytes are marked with black arrows and red arrows, respectively. Mitochondria in the irradiated mice alone are severely edematous with disorganized cristae. Average area **B.** and length (perimeter, **C.**) of the lamellar body in the type II pneumocytes were analyzed in a blind manner using ImageJ software. The histogram represents the mean ± SD of 20 or more lamellar body per group. ^**^P < 0 .01 *vs*. sham group; ^##^*P* < 0.01 *vs*. irradiated group. Scale bar= 0.5 µm.

Lamellar body and mitochondria play important roles in keeping normal alveolar structure and normal physiology function of cells [28]. We, therefore, investigated the protective effects of Ilomastat on the ultrastructures of the lamellar body and mitochondria. Lamellar bodies of type II alveolar epithelial cells in the IR group were atrophic, small, loss of lamellar structure and formed a high electron density solid small clump. The mitochondria exhibited cystic dilatation. Additionally, we also found euchromatin of pneumocytes reduced, while heterochromatin condensed to the periphery around the nucleus, which is the sign of cell apoptosis (Figure 8A, middle). In contrast to the irradiation group, the pneumocytes in sham control and Ilomastat administration groups had normal ultrastructures of the lamellar body and mitochondria (Figure 8A, right and left). The nucleus was also normal (Figure 8A, right and left). The average area of the lamellar body in the radiation group was smaller by 40% than that of in the sham control mice, and by about 20% than that of in the Ilomastat treatment group (Figure 8B). The average perimeter of lamellar body in the radiation group was shorter by 1.5 µm than that of in sham group mice, and by about 1 µm than that of in the Ilomastat treatment group (Figure 8C). These results suggest that Ilomastat may inhibit the breakdown of ultrastructures of the lamellar body and mitochondria.

### Ilomastat pretreatment effectively increases the survival rate of mice

**Figure 9:**
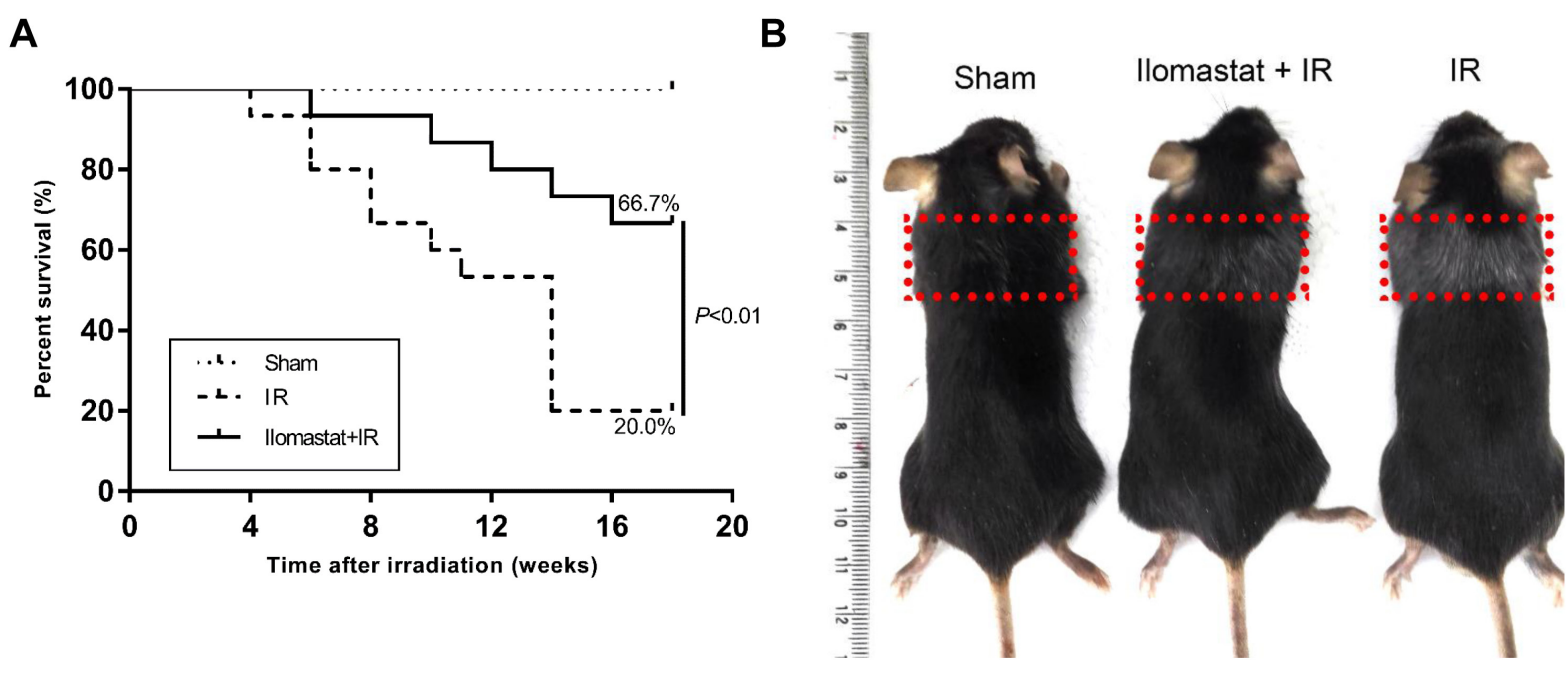
Protective effect of Ilomastat on the survival of mice after irradiation C57BL/6J mice were pretreated with Ilomastat followed by 0 or 15 Gy γ-rays to the whole thorax. Ilomastat pretreatment decreased the death rates of the irradiated mice **A. B.** Representative images of mice treated with 0 Gy, Ilomastat combined irradiation and 15 Gy irradiation alone at 16 weeks.

To investigate the effect of Ilomastat on mouse immortality, we carried out the mice survival experiments. In the irradiation group, 12 of 15 mice (80.0%) died within 16 weeks post-irradiation. However, only 5 of 15 mice in the Ilomastat pretreatment group (33.3%) died within 16 weeks. The survival rates of the sham (*n* = 10), irradiation (*n* = 15) and pretreatment (*n* = 15) groups at the end of the 16^th^ w were 100%, 20.0% and 66.7%, respectively (Figure 9A). The survival rate of the Ilomastat administration group was significantly higher than the irradiation group (*P* < 0.01). Mice exposed to 15Gy irradiation showed more graying coat than Ilomastat administration group in the irradiated region at 16 w (Figure 9B).

### Ilomastat reduces the mitochondria damage in A549 cells and increases the cell survival

**Figure 10:**
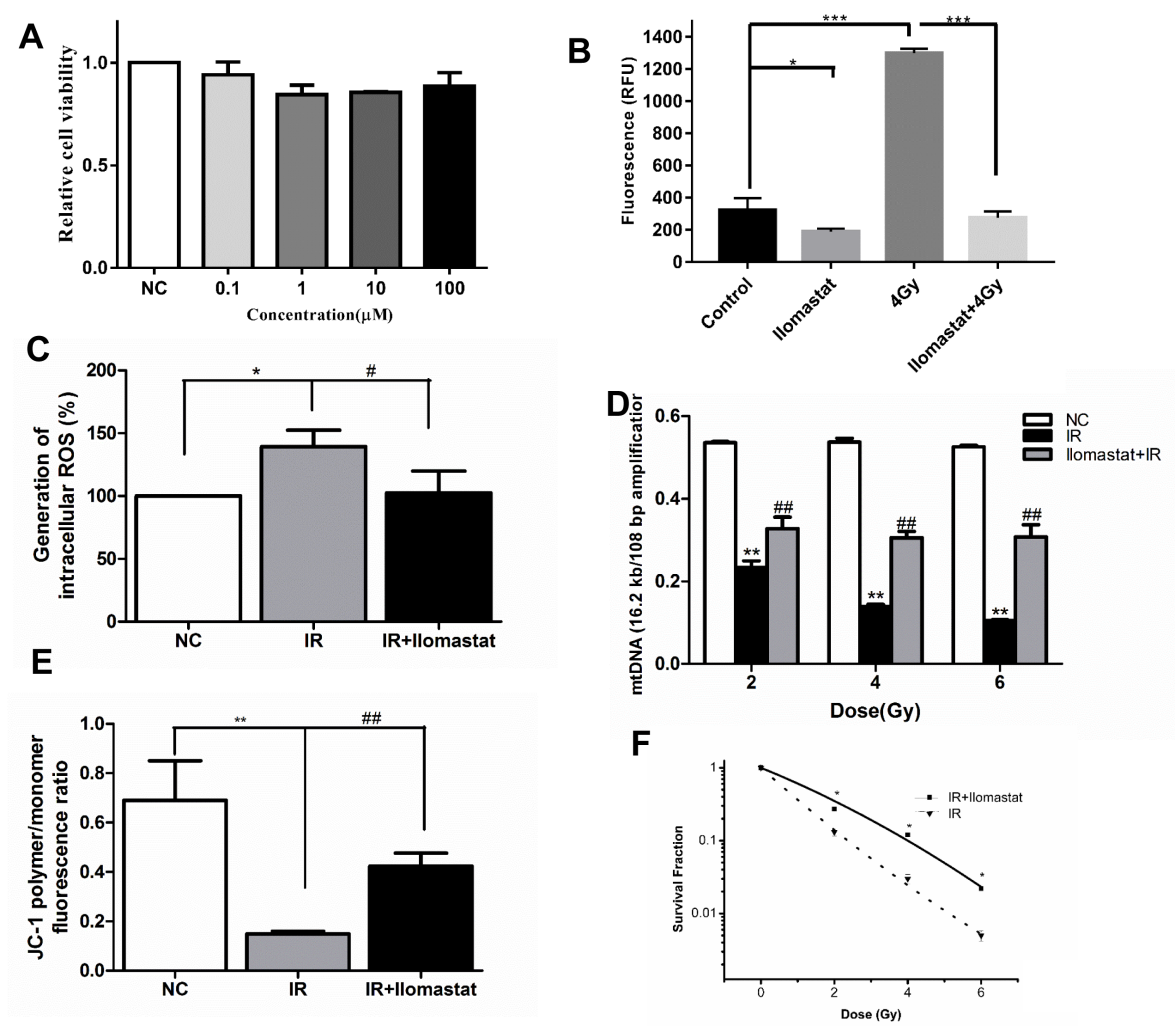
Ilomastat pretreatment reduced radiation induced MMPs activity in A549 cells and the mitochondrial dysfunction **A.** Cells were treated with Ilomastat at various concentrations for 24 h and then the cytotoxicity of Ilomastat were tested by MTT assay. **B.** Assessment of MMP activity in A549 cells pretreated with or without 10 µM Ilomastat following 4 Gy γ-ray irradiation. **C.** Mitochondrial superoxide levels in cells pretreated with or without 10 µM Ilomastat were quantified using fluorescent reagent of DCFH-DA 30 min after 4 Gy γ-ray irradiation. **D.** Damage of mtDNA was determined using mitochondrial genome-specific quantitative extended-length PCR with the KOD FX kit. A549 cells pretreated with or without 10 µM Ilomastat were irradiated with 2, 4 and 6 Gy γ-rays. **E.** Mitochondrial membrane potentials of A549 cells pretreated with or without 10 µM Ilomastat were evaluated using fluorescent reagent of JC-1 6 h after 4 Gy γ-ray irradiation. Numerical data was expressed in terms of the ratio of JC-1 aggregates to JC-1 monomers. **F.** A549 cells pretreated with or without 10 µM Ilomastat were irradiated with 0, 2, 4 and 6 Gy. The colonies were stained by crystal violet 10 days later. Data are representative of three independent experiments and expressed as means ± SD, ^**^P < 0.01 *vs*. sham group; ^#^*P* < 0.05 and ^##^*P* < 0.01 *vs*. irradiated group.

Firstly, we confirmed the cytotoxicity of Ilomastat in different concentration. Our results showed that there had no obvious toxicity till at the concentration of 100 µM (Figure 10A). In addition, we also investigated the MMP activity of cell supernatant after 4 Gy γ-ray irradiation. From our results, we found that irradiation significantly increased the MMP activity, and Ilomastat could decrease the MMP activity to approximately normal level (Figure 10B).

Based on our above experiments, we found that overexpression of MMPs in cells of lung tissue can result in mitochondria dysfunction. Thus, we sought to investigate whether Ilomastat can also alleviate A549 cells mitochondria dysfunction. ROS generation, a biomarker of mitochondria dysfunction, was assessed at 30 min after 4Gy irradiation using DCFH-DA. ROS generation in the single irradiated cells was significantly higher than those of the Ilomastat plus IR treated cells (Figure 10C). Increased ROS generation is known to be involved in the induction of apoptosis through various pathways [29].

From previous research, the accumulation of MMP9 or MMP2 in cells can induce the mtDNA damage [27]. So we sought to investigate this phenomenon could be alleviated by Ilomastat in our cell model. It was found that radiation induced mtDNA damage (almost 40% reduction in the amplification of the long fragment of mtDNA) in a dose-dependent manner (Figure 10D). Ilomastat treatment protected the mtDNA damage induced by IR (Figure 10D).

In healthy cells with high mitochondrial ΔΨm, JC-1 spontaneously forms complexes known as J-aggregates with intense red fluorescence. On the other hand, in apoptotic or unhealthy cells with low ΔΨm, JC-1 remains in the monomeric form, which shows only green fluorescence. The relative proportion of red and green fluorescence is commonly used to measure the degree of mitochondrial depolarization. A decrease in red/green ratio is indicative of apoptosis [30]. Figure 10E shows that Ilomastat pretreatment relieved the apoptosis induced by irradiation (*P* < 0.01). Similarly, Ilomastat pretreatment increased the cell survival, compared to the irradiation group alone (Figure 10F).

These experimental results in cell level also indicate that Ilomastat pretreatmet may attenuate the mitochondrial dysfunction and increase the survival of cells after exposure to irradiation.

## DISCUSSION

RILI is a serious complication after thoracic radiotherapy or nuclear accident and the etiology remains controversial [31]. Its main feature is the pulmonary inflammation and fibrosis. So far, there are no effective treatment and prevention methods. Ilomastat, a most powerful chemical inhibitor of MMPs, has been used in experiment of animal and clinical treatment of tumors in human beings [16, 32]. However, its protective effects on RILI remain unknown. In this study, we verified the protective effects of Ilomastat on RILI in mice and A549 cells, providing a new therapeutic approach to RILI. Our results demonstrate that the inhibitor of MMPs, Ilomastat, ameliorated the irradiated mice from the development of lung inflammation and fibrosis, and improved the survival post-irradiation.

We used a single large dose of radiation (15 Gy) to the entire thorax on the basis of previously reports that this dose induces pneumonitis and diffuse fibrosis in this mouse strain [33, 34]. A lot of dead mice will occur if greater radiation dose is provided to mice, which is not conducive to the subsequent experimental manipulations. However, a lower radiation dose does not produce significant lung injury phenotype, which is also very time-consuming [35].

Rube *et al.* [20] found that there had no influence on the TIMP-1, TIMP-2 and TIMP-3 expression, but increased significantly the MMP2 and MMP9 expression in the lung tissue at 4 and 8 w after 12 Gy irradiation. According to NCBI’s GEO microarray data [35], only MMP9 gene expression was upregulated in the early stage of lung injury of mouse after 17.5 Gy irradiation. Therefore, we investigated the total activity of MMPs in lung homogenate using FRET technique and also detected the expression of MMPs using semi-quantitative RT-PCR. Our results also indicate that irradiation induce the upregulation of MMPs and Ilomastat pretreatment decrease their upregulation. Whereas, as the reported that, at the late stage of RILI, the MMPs’ expressions were very lower in the fibroblasts, immature or mature collagen fibers at the center of the fibrotic foci, and only a few alveolar macrophages and type II pneumocyte retained the expression of the MMPs at the periphery of the fibrotic foci, presumably the advancing front of the fibrosis [4].

MMPs have been proposed as potential therapeutic targets in various pathological conditions involving aberrant MMPs expression and activity. In current study, we found that the mice thorax irradiated by γ-rays led to a progressive alveolitis. The local inflammation of the lung tissues peaked at the end of the 4^th^ w after a single dose γ-ray irradiation, and local lung tissue fibrosis-like changes occurred at the end of the 16^th^ w. Pathological progression from early alveolitis to pulmonary fibrosis is closely associated with the time, which consist with the study of bleomycin-induced lung injury in mice or rats [4]. Wang *et al.* also reported that, eight hours to eight weeks following 15 Gy γ-ray irradiation, pneumonitis was observed in animals and large amounts of collagen depositions were also observed at 16 weeks after the treatment [24]. Whereas, other literatures demonstrated that the pneumonitis at 3-12 weeks and collagen deposition and fibrosis at 24-30 weeks post irradiation were found in the C57BL/6 mice irradiated over their whole thorax with 15 Gy γ-rays [36-38].

MMP9 and MMP2 play key role in degrading type IV collagen, which provides the framework for the basement membrane of pulmonary capillaries, and interstitial collagen and proteoglycan. We found that the mRNA expression of MMP9 and MMP2 peaked at the end of the 1^st^ w and began to decline at the end of the 2^nd^ w. Whereas, Klein *et al.* reported that whole thorax irradiation with 15 Gy induced the expression of MMP2 in the lung at 21 d post irradiation [39]. A possible reason for this difference of the time of MMP9 expression is the different dose rate used in irradiation. As described in previously study, MMP2 and MMP9 secreted by macrophages and neutrophils in bleomycin-induced pulmonary fibrosis in early phase may play an important role in degrading the basement membrane, thereby facilitating the inflammatory cell migration. In mid-phase, parenchymal cells, such as bronchiolar epithelial cells and type II pneumocytes, in addition to the inflammatory cells, also comprise important cellular sources expressing these MMPs. In later phase, when fibrosis progressed, the overall expressions of the MMP2 and MMP9 are very low [4]. Our immunohistochemistry analysis also indicated that the MMP9 protein expressions in irradiation group were significantly greater than the sham control group at the end of the 1^st^, 2^nd^ and 4^th^ w. Although MMP2 mRNA expression increased after radiation, the MMP2 protein expression only slightly increased compared to the control (Figure 2E, low panel). So we did not carry out more analysis for the MMP2 protein expression. The timing of the peak level in previous study [4] is somewhat different from ours. It may be due to the difference of experimental animals used, the different time schedules when animals were sacrificed, or the different treatment manners (for example different radiation styles).

According to the recent studies, mRNA expression of cytokines TGF-β and pro-inflammatory cytokine IL-6 was high in the first several weeks following irradiation [23, 25, 40]. In addition, TGF-β signaling is a potential therapeutic target in lung fibrosis [26]. TGF-β is stored inside the cell and is non-covalently bound to a latency-associated protein (LAP), which keeps TGF-β inactive. The interaction of TGF-β with its receptors to play function requires dissociation of the LAP. Thus, one feasible approach to reducing TGF-β function would be to prevent activation of latent TGF-β. MMP9 and MMP2 proteolytically cleaves latent TGF-β, providing an alternative pathway for activation of latent TGF-β [41]. In current study, after receiving Ilomastat pretreatment combined irradiation, TGF-β and IL-6 expression significantly decreased, and the severity of alveolitis and pulmonary fibrosis reduced in our experiment. Moreover, Ilomastat treatment improved the survival rate of the mice. In light of these results, we speculate that part mechanism of RILI development may be due to the activation of latent TGF-β by MMPs and Ilomastat pretreatment alleviate this progression. IL-6 expression was reported in the bronchial epithelium within a few hours after lung irradiation in C57BL/6J mice, with prolonged release of these cytokines by epithelial and inflammatory cells. Clinical as well as experimental findings have suggested the involvement of IL-6 as a pro-inflammatory cytokine in radiation pneumonia [42, 43]. In our study, we found that the expression of IL-6 increased post-irradiation, and decreased after receiving Ilomastat. However, the in detail mechanism of IL-6 level decreasing remains to be unknown, which needs more experiments to clarify. According to previous research, TNF-α and IL-1β also play vital role in the development of fibrosis [8, 44, 45]. Our results showed that content of TNF-α and IL-1β increased after irradiation. Whereas, Ilomastat pretreatment reduced those two cytokines concentration, which may also contribute to alleviate the lung injury.

Under certain circumstance, activated inflammation cells release superoxide and other ROS, which may activate MMPs [46, 47]. The aggregation of MMPs in the pneumocyte mitochondria is one of the key early events in RILI and MMPs activate the latent TGF-β which further stimulate the inflammation cells release ROS and initiates mitochondrial dysfunction, damages mitochondrial structure, activates apoptotic machinery and damages mitochondrial integrity. Those phenomena also were confirmed by our cell experiment. Inhibition of MMPs could have potential therapeutic value in preventing the continuation of the vicious cycle of mitochondrial damage that the pneumocyte experience in RILI, ultimately inhibiting the development and continued progression of lung inflammation and fibrosis.

There were significant positive correlations of absolute levels between BAL fluid and tissue, and serum and tissue, though there were differences in the peak times. Thus, we used either BALF or tissue homogenate to investigate cytokines change so that we could reduce the amount of experimental mice. In addition, data from genetic mouse models indicate that MMPs knockout may cause enhanced inflammation, intracerebral hemorrhage and brain injury [48]. Thus, inhibition of MMPs activity may require careful test and partial inhibition of MMPs activity may maximize its benefit and minimize the potential toxicity.

In our current study, the survival rate of male C57BL/6 mice after 15 Gy thorax irradiation differed from other results [24, 38, 49]. Dabjan *et al.* reported that the survival data on C57BL/6 mice is most different among the different laboratories because of various radiation dosimetry type and field geometry, mouse age, localized or all lung irradiation, microbiological status, and animal vendor [50]. Of course, we also do not exclude a possibility of the mice status in the period of irradiation. Wang *et al.* have demonstrated that the survival rate at 180-day (about 25 weeks) in the 15 Gy treatment group was 25%.

## CONCLUSIONS

Our experiments demonstrate that the MMPs inhibitor, Ilomastat, effectively alleviate the RILI and suggest that MMPs inhibitor may be used as an alleviator for lung injure induced by acute radiation accident or cancer patient after radiotherapy.

## MATERIALS AND METHODS

### Reagents

5, 5’, 6, 6’-Tetrachloro-1, 1’, 3, 3’-tetraethyl-imidacarbocyanine iodide (JC-1), a fluorescent dye, was obtained from Nanjing Jiancheng Bioengineering Institute (Nanjing, China). 3-(4, 5-Dimethylthiazol-2-yl)-2,5-diphenyltetrazolium bromide (MTT), 2’, 7’-dichlorofluorescin diacetate (DCFH-DA), and N4-hydroxy-N1-[(1S)-1-(1H-indol-3-ylmethyl)-2-(methylamino)-2-oxoethyl]-2-(2-methylpropyl)-, (2R)- (Ilomastat) were obtained from Sigma-Aldrich (St. Louis, MO). All other chemicals and reagents are of analytical grade.

### Animals

Adult male C57BL6 mice (WT mice) (18-22 g, purchased from Laboratory Animal Center, Academy of Military Medical Sciences, Beijing, China, certificate No. SCXK-(Jun)-2012-0004, specific pathogen free) were housed and cared in compliance with the regulations of the NIH and Academy of Military Medical Sciences (Beijing, China). The Committee for Animal Use at the Academy of Military Medical Sciences approved all experimental procedures. To the largest extent, we carried out animal experiment with minimizing the number of animals using as well as lessening their suffering. Water and food were available ad lib in the plastic cages. All mice were acclimated from shipping for 1 week before treatment.

Previous report [5, 51] and our preliminary experiment all showed that Ilomastat has no toxicity on lung tissues in the administration concentration. Thus, in current experiment, the 72 mice were divided into three groups: (i) Sham treatment control group; (ii) mice irradiated with 15 Gy γ-rays only (IR group); (iii) mice pretreated with Ilomastat for 2 h and then radiation with 15 Gy γ-rays (Combined treatment group: Ilomastat plus IR). For survive assay, another 40 mice were treated (10 mice for sham, 15 mice for Ilomastat plus IR and 15 mice for IR only).

### Treatment with the MMP inhibitor Ilomastat

Ilomastat suspension solution was prepared by dissolving it in Tween-80, PEG4000, absolute ethanol and distilled water. Control solutions without Ilomastat were also prepared. The solutions were sterilized by filtration. We prepared a fresh drug solution approximately every 14 d. Animals were injected intraperitoneally once either with 150 mg/kg Ilomastat or vehicle control 2 h before γ-ray radiation.

### Radiation schedule for animal

A ^60^Co irradiator (Model GWXJ80, NPIC, Chengdu, China) was used to conduct γ-ray irradiation. Each mouse for the irradiation was anesthetized with chloral hydrate (50 mg/kg body weight, intraperitoneal injection) and then the whole thorax was locally irradiated at 15 Gy using a ^60^Co source at a dose rate of approximately 95 cGy/min. The field size (2 × 3 cm) was set to provide full exposure of the whole thorax with lead brick-shielding for the remaining parts. The mice were exposed to irradiation one time. The control mice received anesthesia without irradiation.

The mice were anesthetized and confined in specifically designed jigs and placed so that the thoraces were in the field of radiation. Following irradiation, the mice were maintained four to six per cage and supplied with standard laboratory chow and water ad lib. Age-matched controls were maintained under identical conditions for the course of the experiment. At the end of 1^st^, 2^nd^, 4^th^ and 16^th^ w the mouse were sacrificed for the further experiments.

### Bronchoalveolar lavage fluid (BALF) preparation

Three to five mice from each group after irradiation were euthanized by chloral hydrate at the end of 1^st^, 2^nd^, 4^th^ and 16^th^ w after the irradiation. Lungs were flushed 3 times with 0.5 ml ice cold PBS-EDTA (1 × PBS, 0.2% EDTA) and these solutions from each lung were pooled.

### Measurement of protease activity

Concentration of protein extracts were measured with a BCA assay kit. Total activity of MMPs was determined using a fluorogenic substrate (5 μM): Mca-Lys-Pro-Leu-Gly-Leu-Dap-Ala-Arg-NH2 (Mca-KPLGL-Dap (Dnp)-AR-NH_2,_ Chinese Peptide Company, Hangzhou, China). Briefly, Certain content of protein extracts and fluorogenic substrate were incubated for 2 h at 37°C in 0.1 M Tris-HCl assay buffer (pH 7.5) containing 150 mM NaCl, 10 mM CaCl_2_, 0.1 mM ZnCl_2_, 0.05% (w/v) Brij35, 0.1% (w/v) PEG6000 [52, 53]. The fluoresence intensity of protease activity were measured at a fluorescence excitation wavelength 328 nm and an emission wavelength of 400 nm using a fluorescence plate reader (SpectraMax M5 Microplate Readers; Molecular Devices, Sunnyvale, CA).

### Semi-quantitative RT-PCR and quantitative real-time polymerase chain reaction (qRT-PCR) analysis for mRNA expression

The whole lungs were immediately removed after death without being perfused, flash frozen in liquid N_2_, and homogenized in buffer RZ (TIANGEN, Beijing, China). The rest procedures were carried out according to the manufacturer’s instructions. For RT-PCR, reverse transcription was carried out with GoScript™ Reverse Transcription System (Promega, USA). Two micrograms of total RNA were reverse transcribed to cDNA as previously described [26]. Five times diluted cDNA product was used in the PCR. The primers of MMP9, MMP2, TIMP-1, TIMP-2 and internal control of β-actin were synthesized by SBS Genetech, and the PCR primers for mouse were listed in Table 1. cDNA (1µL) was amplified with 2 × Taq DNA polymerase (TIANGEN, Beijing, China), and PCR cycle was as follows: an initial denaturation was performed at 94°C for 2 min; then 94°C 30 s, 60°C 30 s and 72°C 30 s repeated for 35 cycles. The PCR products then were resolved on 1.5% agarose gel, with a DL1000 DNA ladder (standard) run simultaneously on each gel. Relative amplification was quantified by normalizing the gene-specific band intensity to that of β-actin. Quantitative analysis was achieved by measuring the integrated density value of PCR products in gel photographs using Gel-pro analysis software (vision 4.0). For qRT-PCR, samples were performed using a Light Cycler^®^ 96 (Roche Diagnostics). All steps were carried out according to the protocol. The relative fold change of mRNA was calculated by using the 2^-ΔΔCt^ method.

**Table 1:** Sequences of PCR primers used in this study

Primers	Sequences(5’-3’)
MMP9 forward	AAAGGCAGCGTTAGCCAGAA
MMP9 reverse	GGTCTTTGGGGAAGACCACA
MMP2 forward	AACGGTCGGGAATACAGCAG
MMP2 reverse	GTAAACAAGGCTTCATGGGGG
TIMP-1 forward	GGCATCTGGCATCCTCTTGT
TIMP-1 reverse	TGGTCTCGTTGATTTCTGGGG
TIMP-2 forward	CAGCCTCTCCCGTCTTTTGT
TIMP-2 reverse	GTGGCTAGAAACCCCAGCAT
β-actin forward	CACTGTCGAGTCGCGTCC
β-actin reverse	CGCAGCGATATCGTCATCCA

### Tissue isolation and histological staining

Three to five mice from each group were sacrificed for lung histological analysis. After anesthesia the left lungs of the mice were perfused in situ via the trachea with 4% paraformaldehyde solution. The lungs were subsequently removed and placed in fixative as described previously [24, 49]. The fixed lung tissue was processed into paraffin and sections of 3 μm in thickness were prepared for analysis from the midhorizontal sections of the lung lobes encompassing the largest surface area. Sections were stained by hematoxylin and eosin (H&E) to examine general tissue morphology and by Masson’s trichrome staining to visualize the connective tissue (collagen fibers). The degrees of pulmonary fibrosis were assessed in a blind manner by examining random sections at 100× magnification, according to an established grading scales from 0 (normal lung) to 8 (severe distortion of structure) [54, 55]. Then Image Pro-Plus 6.0 software for image analysis was used to calculate area ratio (fibrosis area/total field area) to evaluate the degree of mice pulmonary fibrosis. The extent of the alveolitis was graded on a scale of 0 (normal) to 3 (severe) [24, 56]. For the grading of alveolitis, each group was comprised of three to five animals. In each animal, a section was obtained from the middle lobe of the right lung, and alveolitis was graded out of five microscopic fields in each tissue section.

### Cytokine assay

Transforming growth factor-β (TGF-β) and interleukin-6 (IL-6) Enzyme-Linked Immunosorbent Assay (ELISA) kits were purchased from Wuhan Boster Biological Technology Co.Ltd (Wuhan, China). Mouse tumor necrosis factor-α (TNF-α) and interleukin-1 beta (IL-1β) ELISA kit was purchased from Beijing Dakewe Biotechnology, Inc (Beijing, China). Assay of cytokines were performed with ELISA kits according to the manufacturer’s protocol.

### Immunohistochemistry

All tissue sections were stained according to the manufacturer’s instructions. Heat induced epitope retrieval was performed and a standard DAB detection kit was used for visualization of the immunoreaction (DAKO, Denmark). MMP9 and MMP2 expression were detected using a rabbit anti-mouse MMP9 and MMP2 polyclonal antibody (sc-393859 and sc-53630; Santa Cruz Biotechnology) diluted in 1:100. MPO and F4/80 expression were detected using a rabbit anti-mouse MPO and F4/80 polyclonal antibody (GB13027, GB11224; Servicebio, Wuhan, China), diluted in 1:800 and 1:2000, respectively. Sections were incubated at 4°C overnight with the primary antibodies and with biotinylated goat anti-rabbit IgG (KPL, Burlingame, CA) as secondary antibodies for 50 min at room temperature. Negative controls subjected to the same procedure without the primary antibody showed consistently negative results. MPO or F4/80-positive cells were counted by randomly selecting five high-power fields distributed over at least three independent sections.

The relative expressions of MMP9 and MMP2 proteins were quantified using the image-pro-plus 6.0 software (Media Cybernetics Company, USA). Five fields in each section were randomly chosen. The fraction of total intensity of brown substances (positive staining)/total area in each image (field) indicates the relative expression of the corresponding protein.

### Measurement of lung hydroxyproline

Collagen deposition was evaluated by detecting the total hydroxyproline content of the lungs using a Mouse Hydroxyproline Assay Kit from Nanjing Jiancheng Bioengineering institute according to the manufacturer’s protocols. Samples of lung tissue from mice were weighed about 30-100 mg in a test tube and added accurately hydrolysate 1 mL. Tubes were capped and heated with boiling water bath for 20 min. The solutions were cooling to room temperature and adjusted to 6.0-6.8 with a pH adjusting agents. Diluted hydrolysates were added suitable activated carbon. The hydrolysates were centrifuged at 3500 rpm for 10 min and then were incubated for 90 min at 60°C and measured the absorbance at 550 nm (A550). Hydroxyproline standard needed to be measured as positive control. The background for the assay represents the value obtained for the ultrapure water. The results were presented as micrograms per gram of lung tissues.

### Lamellar bodies and mitochondrial morphology of the type II pneumocyte

Ultrastructures of the lamellar bodies and mitochondria in the lung were evaluated by transmission electron microscopy (TEM). The lung was cut into 1mm^3^ pieces and pre-fixed for 24 h in 3% glutaraldehyde (in 75 mM phosphate buffer, pH = 7.4), and then was rinsed in 75 mM phosphate buffer with 0.19 M sucrose buffer for several times at 4°C. The treated lung was post-fixed in 1% buffered OsO_4_ (in 240 mM phosphate buffer, pH = 7.4) at 4°C and rinsed in 75 mM phosphate buffer with 0.19M sucrose buffer for 15min at 4°C.The lung then was dehydrated using graded ethanol solutions. After soaking the samples in 100% acetone and embedding medium (v/v=1:1) at room temperature for 30min and pure embedding medium overnight, the lung pieces was embedded following the polymerization procedure, 35°C for 12 h, 45°C for 12 h and 60°C for 24 h. Ultrathin sections (60-70 nm) of the lung then were prepared. The sections were stained with uranyl acetate and lead citrate for 10 min, respectively. The sections were viewed by TEM (H-7650, Hitachi, Japan). At least eight random images were recorded from each independent preparation and enlarged to 20,000 × magnification. Lamellar bodies of the type II pneumocyte were analyzed in a blind manner by ImageJ software to calculate their area (mm^2^) and perimeter length (mm).

### TUNEL assay apoptosis of pneumocyte

The TUNEL assay [27] was performed to evaluate the apoptosis of pneumocyte using the *in situ* cell death detection kit, POD (Roche Molecular Biochemicals, Indianapolis, IN) according to the manufacturer’s instructions. Briefly, slides were deparaffinized in xylene and rehydrated in graded ethanol, and the protein was digested with 20 µg/mL proteinase K for 30 min at 37°C incubator. The slides were washed with two changes of phosphate buffered saline (PBS). After being covered with permeabilisation solution (0.1% TritonX-100, 0.1% sodium citrate, freshly prepared), the slides were washed with two changes of phosphate buffered saline (PBS). After that, terminal deoxyribonucleotidyl transferase (TdT) enzyme and dUTP were mixed at a 2:29 volume rate and incubated for 2 h at 37°C in humidified chamber. The reaction was then stopped using the blocking buffer provided and the slides were rinsed three times with PBS. The provided converter-POD was applied for 30 min at 37°C. After being washed with PBS, the slides were treated with DAB substrate. The reaction time was estimated through light microscopy until the nucleus of positive cells display brown. The reaction was stopped with running water when positive cells were stained with brown nucleus. The slides were counterstained with hematoxylin at a matter of 3 min.

### Cell culture

A549 cells, a tumor cell line from a human lung carcinoma with properties of type II alveolar epithelial cells, were purchased from American Type Culture Collection (ATCC). This cell line has been used as a model of human type II alveolar epithelial cells in the literature [57]. The cells were grown in 60-mm tissue culture dishes in a humidified atmosphere of 5% CO_2_. The culture medium was RPMI-1640 (Gibco, USA) containing 10% heat-inactivated fetal calf serum (Hyclone, USA), and 1% penicillin/streptomycin. When the cells reached confluence, the culture media were changed to the serum-free defined medium of the above-mentioned composition except for heat-inactivated fetal calf serum.

### Cytotoxicity assay

Cells were seeded at a density of 1×10^4^ cells/well in a 96-well plate and grown overnight. Cells were treated with different concentration Ilomastat (0-100 µM) diluted by 100 μL of fresh medium, and then were incubated for 24 h at 37°C. 10 μL of MTT solution (5mg/ml) was added to each well. The cells were incubated at 37°C for 4 h. Absorbance was recorded at 490 nm by a microplate reader (SpectraMax M5, Molecular Devices, USA).

### Cell irradiation

After incubation for 2 h with or without Ilomastat (10 µM), the cells were irradiated at room temperature with different doses of γ-rays from ^60^Co source (dose rate of 93.2 cGy/min). The conditioned media were collected 24 h after irradiation.

### Mitochondrial damage analysis in A549 cells

ROS level was determined using a fluorescent probe, 2, 7, dichlorodihydrofluorescein diacetate (DCFH-DA). Cells were seeded in 6-well plates with growth medium. The media were removed, and the cells were incubated with 5 μM of DCFH-DA in the growth medium for 30 min at 37 °C and 5 % CO_2_. Images were taken using fluorescence microscope (Olympus, Japan).

The collapse of mitochondrial membrane potential (ΔΨm) was investigated by using a molecular Probes JC-1. At the designed time points, the cells were washed with PBS and incubated with 2 μM of JC-1 dye in PBS (pH = 7.4) at 37°C in the dark for 20 min. The images were taken by inverted fluorescent microscope (Olympus, Japan) and the mitochondrial depolarization patterns of cells for quantification were examined using imaging software Image J.

Mitochondrial DNA damage was determined by mitochondrial genome-specific quantitative extended-length PCR with the KOD FX kit (TOYOBO, Japan). In brief, 15 ng DNA was amplified by using genome-specific primers (mtDNA-long, forward 5’-TGAGGCCAAATATCATTCTGAGGGGC-3’ and reverse 5’-TTTCATCATGCGGAGATGTTGGATGG-3’; mtDNA-short, forward 5’-CACCCAAGAACAGGGTTTGT-3’ and reverse 5’-TGGCCATGGGTATGTTGTTAA-3’ and 1 unit KOD FX DNA polymerase. Mitochondrial DNA was amplified with 24 cycles, and PCR products were resolved on agarose gel. Relative amplification was quantified by normalizing the intensity of the long product to the short product (mtDNA = 16.2 kb/108 bp).

### Clonogenic survival assay

After irradiation, cells were washed with PBS buffer and trypsinized. An appropriate number of cells were plated into each 60-mm dish to produce colonies. After incubating for 10 d, the cells were fixed with 10 mL fresh Carnoy’s fluid, stained with 0.5% crystal violet for 20 min. The number of colonies with greater than 50 cells was counted as survivors. Plating efficiencies (PE) were calculated as follows: numbers of colonies formed/numbers of cells plated. Surviving fractions were calculated as follows: PE (irradiated)/PE (unirradiated). All experiments were performed in triplicate. The experiment was at least repeated for three times independently.

### Statistical analysis

All data are presented as means ± standard deviation (SD). One-way Analysis of Variance (ANOVA) test were used to compare the significance of numerical data between groups utilizing Graph Pad Prism^®^ software (vision 5.0). The Kaplan-Meier method was used to analyze the mouse survival. Comparison of categorical data between two groups was performed with chi-square test for animal experiment and student’s t test for the cellular level experiment. Values were considered statistically significant when *P*-value less than 0.05. All statistical tests were two-tailed.

## Abbreviations

RILI: radiation induced lung injury
w: week
H&E staining: hematoxylin and eosin staining
MMPs: matrix metalloproteinases
MMP9: matrix metalloproteinase 9
MMP2: matrix metalloproteinase 2
IR: irradiation
γ-ray: gamma-ray
Gy: gray
TGF- β: transforming growth factor-β
IL-6: interleukin 6
IL-1β: interleukin 1 beta
TNF-α: tumor necrosis factor-α
RLU: relative light units
TBS: Tris-buffered saline
TIMP-1: tissue inhibitor of metalloproteinases-1
TIMP-2: tissue inhibitor of metalloproteinases-2
LAP: latency-associated protein
PCR: polymerase chain reaction
RT-PCR: reverse transcription PCR
TUNEL: terminal deoxyuridine nick-end labeling
ROS: reactive oxygen species
PBS: phosphate buffered saline
BALF: bronchoalveolar lavage fluid
COPD: chronic obstructive pulmonary disease
MS: multiple sclerosis
ELISA: enzyme linked immunosorbent assay
